# Orthotopic hepatocellular carcinoma: molecular imaging-monitored intratumoral hyperthermia-enhanced direct oncolytic virotherapy

**DOI:** 10.1080/02656736.2019.1569731

**Published:** 2019-02-18

**Authors:** Jingjing Song, Feng Zhang, Jiansong Ji, Minjiang Chen, Qiang Li, Qiaoyou Weng, Shannon Gu, Matthew J. Kogut, Xiaoming Yang

**Affiliations:** a Image-Guided Bio-Molecular Interventions Section, Division of Interventional Radiology, Department of Radiology, University of Washington School of Medicine, Seattle, WA, USA; b Key Laboratory of Imaging Diagnosis and Minimally Invasive Intervention Research, The Affiliated Lishui Hospital of Zhejiang University, The Fifth Affiliated Hospital of Wenzhou Medical University, Lishui Central Hospital, Zhejiang, China; c Department of Radiology, Yinzhou People’s Hospital Ningbo, Ningbo, Zhejiang, China; d Department of Radiology, Sir Run Run Shaw Hospital, Zhejiang University School of Medicine, Hangzhou, Zhejiang, China

**Keywords:** Molecular imaging, radiofrequency hyperthermia, oncolytic virus, hepatocellular carcinoma

## Abstract

**Objective:**

To validate the feasibility of molecular imaging-monitored intratumoral radiofrequency hyperthermia (RFH) enhanced direct oncolytic virotherapy for hepatocellular carcinoma (HCC).

**Methods:**

This study included *in vitro* experiments using luciferase-labeled rat HCC cells and *in vivo* validation experiments on rat models with orthotopic HCCs. Both cells and HCCs in four groups (*n* = 6/group) were treated by: (1) combination therapy of oncolytic virotherapy (T-VEC) plus RFH at 42 °C for 30 min; (2) oncolytic virotherapy alone; (3) RFH alone; and (4) saline. For *in vitro* confirmation, confocal microscopy and bioluminescence optical imaging were used to evaluate the cell viabilities. For *in vivo* validation, oncolytic viruses were directly infused into rat HCCs through a multi-functional perfusion-thermal RF electrode, followed by RFH. Ultrasound and optical imaging were used to follow up size and bioluminescence signal changes of tumors overtime, which were correlated with subsequent laboratory examinations.

**Results:**

For *in vitro* experiments, confocal microscopy showed the lowest number of viable cells, as well as a significant decrease of bioluminescence signal intensity of cells with combination therapy group, compared to other three groups (*p* < .001). For *in vivo* experiments, ultrasound and optical imaging showed the smallest tumor volume, and significantly decreased bioluminescence signal intensity in combination therapy group compared to other three groups (*p* < .05), which were well correlated with pathologic analysis.

**Conclusion:**

It is feasible of using molecular imaging to guide RFH-enhanced intratumoral oncolytic virotherapy of HCC, which may open new avenues to prevent residual or recurrent disease of thermally ablated intermediate-to-large HCCs.

## Introduction

Hepatocellular carcinoma (HCC) is the sixth most common malignancy worldwide and the third most common cause of cancer-related death [1]. Although hepatic resection and transplantation are considered as the curative therapy for early-stage HCC, only 20% of HCC patients are candidates for resection due to decompensated cirrhosis, portal hypertension, or various comorbidities [2,3]. Liver transplantation is precluded for a majority of patients because of the shortage of liver donors [4,5]. Radiofrequency ablation (RFA) is the most effective locoregional treatment of early-stage HCC, with outcomes similar to resection for HCC tumors up to 3 cm in diameter [6–11]. Nonetheless, local recurrence at the margin of intermediate (3–5 cm) and large (5–7 cm) HCC tumors after RFA remains a pitfall [12]. Therefore, there is a pressing demand to develop techniques to improve local control after RFA of larger HCC tumors. Although combined transarterial chemoembolization (TACE) and RFA has shown improved efficacy compared to RFA alone, the local tumor progression rate in large tumors is still up to 32% after the combination treatment [13].

Oncolytic virus immunotherapy is a fast developing and promising therapeutic approach that utilizes native or genetically modified viruses to selectively infect, replicate within, and thereby lyse cancer cells without harming normal tissues [14–17]. The distinct mechanisms of oncolytic viruses that mediate the antitumor activity are thought to be associated with the selective replication of virus within neoplastic cells, which results in a direct lytic effect on tumor cells and the induction of systemic antitumor immunity [14].

To date, many oncolytic viruses have been developed to treat human cancers in early-phase clinical trials, such as adenoviruses, poxviruses, herpes simplex virus-1 (HSV-1), coxsackie viruses, poliovirus, measles virus, newcastle disease virus (NDV), reovirus [14]. Talimogene laherparepvec (T-VEC), a HSV-1-derived and genetically-modified oncolytic virus, is the first one that has been approved by US Food and Drug Administration (FDA) for treating advanced melanoma [16]. A randomized Phase III clinical trial showed an improved and durable tumor response in patients treated with systemic administration of T-VEC for their advanced melanoma [14].

Our previous studies have confirmed that image-guided radiofrequency hyperthermia could increase the uptake of intratumoral chemotherapy in cancer cells and thus significantly enhance the effect chemotherapy on HCC [18–20]. This groundwork led to our current study design, combining intratumoral administration of T-VEC-mediated virotherapy with the same session of radiofrequency hyperthermia (RFH). The purpose of this study was to determine the feasibility of using intratumoral RFH to enhance the direct oncolytic virotherapy of orthotopic HCC in rats, which was monitored by molecular imaging.

## Materials and methods

### Study design

Our study was divided into two stages: (a) serial *in vitro* experiments to confirm that RFH could enhance the therapeutic effect of T-VEC on rat HCC cells (McA-RH 7777, ATCC, Manassas, Virginia); (b) serial *in vivo* experiments to validate the feasibility that RFH combined with intratumoral oncolytic virotherapy has the synergistic therapeutic effect on rat models with orthotopic HCCs. Observers were blinded to the animals performed.

### In vitro evaluation

#### Cell culture and RFH-enhanced killing effect of T-VEC

McA-RH7777 cells were transduced with luciferase/red fluorescence protein/lentivirus to create luciferase and red fluorescence protein positive HCC cells according to the manufacturer’s protocol (GeneCopoeia, Rockville, Md). Luciferase and red fluorescence protein positive cells were sorted by using a fluorescence-activated cell sorting technique (Aria II, Becton Dickinson, Franklin Lakes, NJ). Cells were then seeded in four-chamber cell culture slides (Nalge Nunc International, Rochester, NY) and maintained in Dulbecco’s Modified Eagle’s Medium (DMEM, Life Technologies, Carlsbad, CA) supplemented with 10% fetal bovine serum (Gibco, Grand island, NY). RFH was performed by placing a .022-inch MR imaging heating guidewire under the bottom of chamber four of the chamber slides. A 400-μm fiber optical temperature probe (Photon Control, Burnaby, British Columbia, Canada) was placed in the chamber for temperature measurement. By adjusting RF output power at approximately 10 W, the temperature of chamber four was kept at 42 °C, while chamber one at 37 °C [21]. Cells in different groups were treated with (1) T-VEC (MOI = 0.2) plus 30 min of RFH at 42 °C; (2) T-VEC (MOI = 0.2) alone; (3) 30 min of RFH at 42 °C alone; (4) saline to serve as a control group. We used the 50% inhibitory concentration (ID, infectious dose 50) doses of T-VEC for cell treatment, which were determined by means of MTS assay (3-[4, 5-dimethylthiazol-2-yl] II-5-[3-carboxymethoxyphenyl]-2-[4-sulfophenyl]-2H-tetrazoliu, CellTiter 96 Aqueous One Solution Cell Proliferation Assay; Promega, Madison, WI). All experiments for each of the cell groups were repeated six times.

#### Cell proliferation assay

Cells proliferation was evaluated by MTS assay 24 h after the treatments. Relative cell proliferations of different cell groups were calculated by using the equation of *A*_treated_ – *A*_blank_/*A*_control_ – *A*_blank_, where *A* is absorbance. Cells on cell culture slides were subsequently washed twice with PBS (phosphate buffered saline), fixed in 4% paraformaldehyde, counter-stained with 4′,6-diamidino-2-phenylindole (DAPI; Vector Laboratories, Burlingame, CA), and then imaged with a fluorescence microscope [22,23].

#### Apoptosis assay

The percentages of viable as well as apoptotic cells were quantified by flow cytometry using Annexin V-fluorescein isothiocyanate and propidium iodide (PI) staining (BD Biosciences, San Diego, CA). Cells were stained with Annexin-V/FITC and PI in a binding buffer along with the appropriate control. Total number of Annexin V and PI positive cells were counted using a FACScan flow cytometer (BD Biosciences). The data was analyzed using software (FlowJo version 10; FloJo Data Analysis Software, Ashland, OR).

#### Bioluminescence optical imaging of treated cells

Fluorescent signal intensity was also evaluated 24 h after the treatments. Cells in four groups were collected and suspended in 50 μL saline. Subsequently, 5 μL of Pierce d-Luciferin (ThermoFisher Scientific, Rockford, IL) was added into the cells suspension and incubated for an additional 20 min. The cells suspension was transferred to cylindrical glass tubes and mixed with 50 μL 1% agarose. Bioluminescence optical imaging was performed for the cells using an *in vivo* imaging system (In Vivo Imaging, Bruker Corp., USA). Bioluminescence signal intensity was quantified as the sum of all detected photon counts. Data were normalized to relative signal intensity (RSI) by using the following equation: RSI = SI_T_/SI_C_, where *SI* is signal intensity, *T* represents the treatment group, and *C* represents the control group.

### In vivo confirmation

#### Creation of animal models

The animal protocol was approved by our institutional animal care and use committee. Nude rats (RNU Rat, Charles River, Skokie, IL) were anesthetized with 1–3% isoflurane (Piramal Healthcare, Andhra Pradesh, India) in 100% oxygen. Twenty-four rats, weighed 180–220 g, were used for creation of rat models with orthotopic HCCs. The rats were positioned supine on the surgical table. A sterile laparotomy was performed to expose the left lobe of the liver, followed by an injection of 0.5–1 × 10^7^ luciferase positive McA-RH7777 cells in 100 μL of saline into the liver parenchyma, 5-min compression of cell injection site with a small piece of gelatin sponge (Pharmacia & Upjohn Co, Division of Pfizer Inc, NY), and the closure of the abdominal incision with layered sutures. Ultrasound and bioluminescence optical imaging were used to follow up the growth of rat orthotopic HCCs, until the tumors reached to 8–10 mm in diameter. The twenty-four rats with orthotopic HCCs were randomly allocated into four groups. Six rats in each of the four groups were treated with (1) T-VEC plus 30 min of RFH at 42 °C; (2) T-VEC alone; (3) 30 min of RFH at 42 °C alone; (4) saline to serve as a control group.

#### RFH-enhanced direct oncolytic virotherapy of orthotopic HCCs

**Figure 1.**
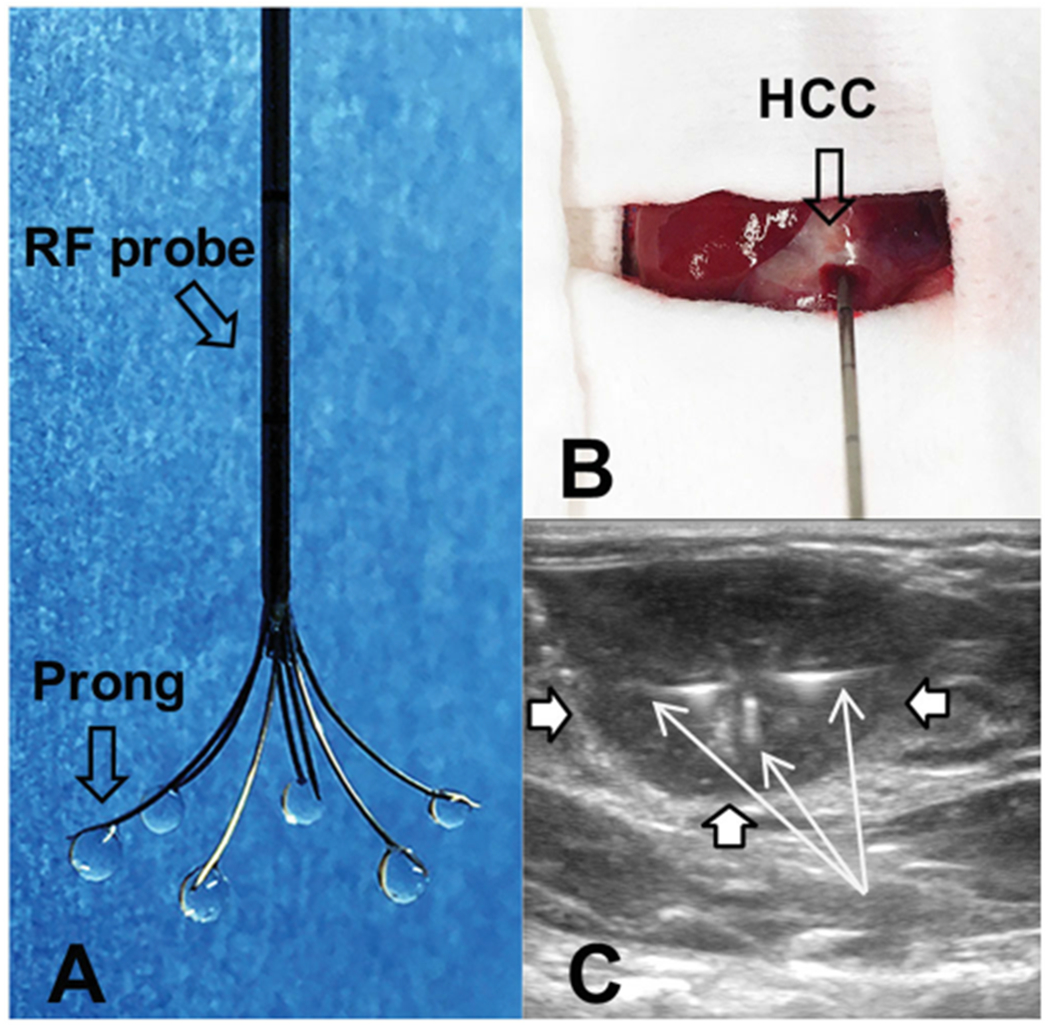
(A) The photograph shows the multi-functional perfusion-thermal electrode, with multi-prongs for delivery of oncolytic viruses and thermal energy simultaneously. (B) The RF electrode is positioned in the center of a rat hepatic HCC (open arrow) under ultrasound guidance. (C). Oncolytic viruses are locally delivered into the tumor margin through the infusion prongs (arrows), where RF hyperthermia is simultaneously generated to further enhance the uptake of oncolytic viruses by the HCC (open arrows) cells.

We used a multi-functional perfusion-thermal RF electrode (Welfare Electronics Co., Beijing), which consists of multiple prongs. Each prong has an infusion channel and thermal sensor at its tip (Figure 1(A)). Via laparotomy, the RF electrode was precisely positioned in the center of the tumor under ultrasound guidance (Sonosite; Bothell, WA). The array of the multiple prongs was opened to cover the periphery of the tumor, where 10^6^ pfu T-VEC oncolytic virus in 100 μL saline was directly infused within 3 min (Figure 1(B,C)). Immediately after T-VEC oncolytic virus delivery, RFH was delivered to the tumor by using a radiofrequency ablation system (We7568-II, Welfare Electronics Co.). By adjusting RF output power at approximately 30 W, the temperature at the tumor periphery was kept at 42 °C for 30 min.

#### Post-treatment follow-up with US and optical imaging

US imaging was performed to assess the tumor growth at days 0, 7, and 14 after the treatment. The axial (*x*) and longitudinal (*y*) diameters of tumors and tumor depths (*z*) were measured on the US images at the largest dimension. The volume of each tumor was then calculated according to the following equation: *v* = *x* · *y* · *z* · *π*/6. Data were expressed as relative tumor volume (RTV) by using the following equation: RTV = *V_D_n__/V*_*D*_0__, where *V* is tumor volume, *D_n_* represents days after treatments, and *D*_0_ is the day before treatment.

wIn addition, we also used optical imaging to follow up tumor response to the treatments. Optical imaging was conducted using the *in vivo* imaging system (Bruker). Each animal was imaged at days 0, 7, and 14. Optical images were acquired 20 min immediately after an intraperitoneal administration of d-luciferin at 150 mg/kg (Pierce d-Luciferin; ThermoFisher Scientific, Rockford, III). Signal intensity of the tumor was quantified by using the Bruker software. Relative signal intensity (RSI) was calculated by using the following equation: RSI = SI*_D_n__*/SI_*D*_0__, where SI is signal intensity, *D_n_* represents days after treatment, and *D*_0_ is the day before treatment.

#### Pathologic correlation/confirmation

Tumors were harvested at day 14 after treatment. Tumor tissues were fixed with 10% formalin for 4 h and then embedded in paraffin. Hematoxylin and Eosin (H & E) staining was performed to confirm the formation of the tumors, and terminal deoxynucleotidyl transferase dUTP nick end labeling (TUNEL) assay was carried out according to the manufacturer’s protocol (Roche Molecular Biochemicals, Mannheim, Germany) to evaluate the apoptosis. A brown or tan-colored staining of cells indicated the apoptotic cells. The apoptosis level of tumors was expressed as apoptotic index, defined as the number of apoptotic cells/total number of cells × 100%. At least 1000 cells from 10 fields at the magnification of 20× were counted using the DMRCQ550 system (Olympus, Tokyo, Japan) technology.

### Statistical analysis

Statistical software (SPSS 19.0; SPSS, Chicago, IL) was used for all data analyses. The non-parametric Mann-Whitney *U* test was used to compare (a) relative proliferation rates among different cell groups; (b) relative optical signal intensities as well as (c) relative tumor volumes at different time points among the animal groups with various treatments. The data were reported as mean ± standard error. A *p* values of less than .05 was considered statistically significant.

## Results

### In vitro evaluation: RFH-enhanced killing effect of T-VEC on HCC cells

**Figure 2.**
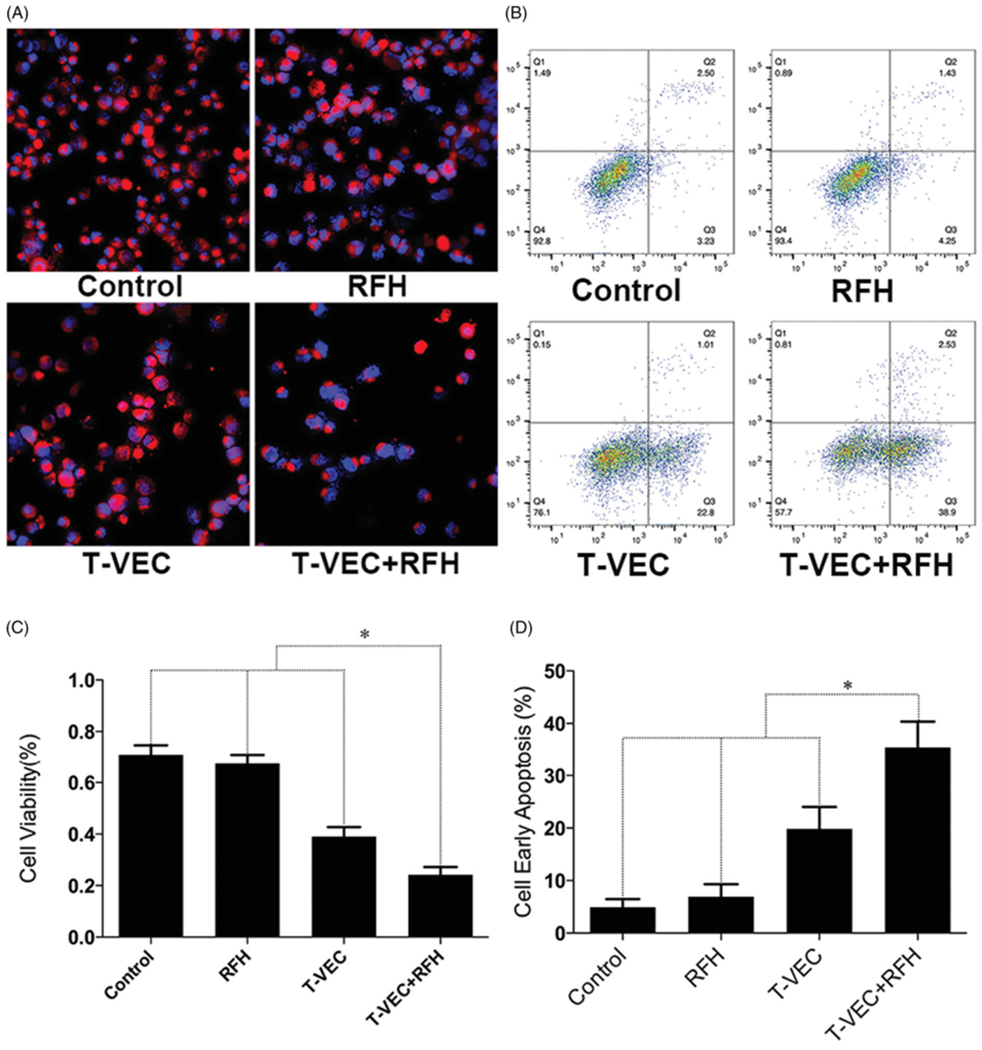
(A) Confocal microscopy shows the lowest number of viable cells in the group treated with combination therapy, compared with other three groups. (B) MTS assay further demonstrates the lowest cell viability in combination therapy group (*p<.001). (C&D) Flow cytometry shows the highest percentage of early apoptotic HCC cells in combination therapy group, compared with other three groups (*p<.001).

Confocal microscopy performed 24 h after treatments demonstrated diminished cell survival with the combination therapy group, compared with those of the other three treatment groups (Figure 2(A)). Quantitative MTS assay further quantitatively confirmed that cell proliferation with combination therapy was significantly lower than those in the groups treated with T-VEC therapy alone, RFH alone, and saline (relative absorbance of formazan: 24.2 ± 3.0% vs 39.1 ± 3.7%, 67.5 ± 3.4%, 70.9 ± 3.7%, *p*<.001) (Figure 2(B)). Apoptosis analysis by flow cytometry further demonstrated more apoptotic cells in the combination therapy group than the other three groups (35.4 ± 4.9% vs 19.8 ± 4.2% vs 6.9 ± 2.4% vs 5.0 ± 1.5%, respectively, *p* < .001) (Figure 2(C,D)).

**Figure 3.**
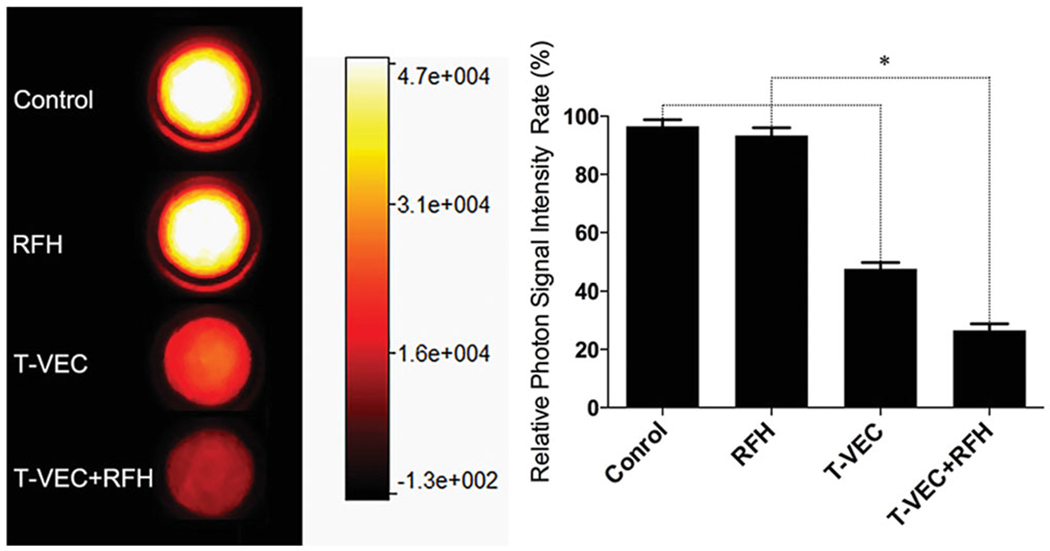
Fluorescent signal intensity is quantified 24 h after the treatments. There is a significant decrease in relative fluorescent signal (golden-yellow color) with combination therapy group, compared to other three treatments (*p<.001).

Bioluminescence optical imaging showed a significant decrease in relative bioluminescence signal with combination therapy, compared to the other three treatment groups (0.27 ± 0.02 × 10^7^ vs 0.48 ± 0.02 × 10^7^ vs 0.93 ± 0.03 × 10^7^ vs 0.97 ± 0.02 × 10^7^, respectively, *P* < .001) (Figure 3).

### In vivo *confirmation: Intrahepatic RFH-enhanced oncolytic virotherapy of orthotopic HCCs*

**Figure 4.**
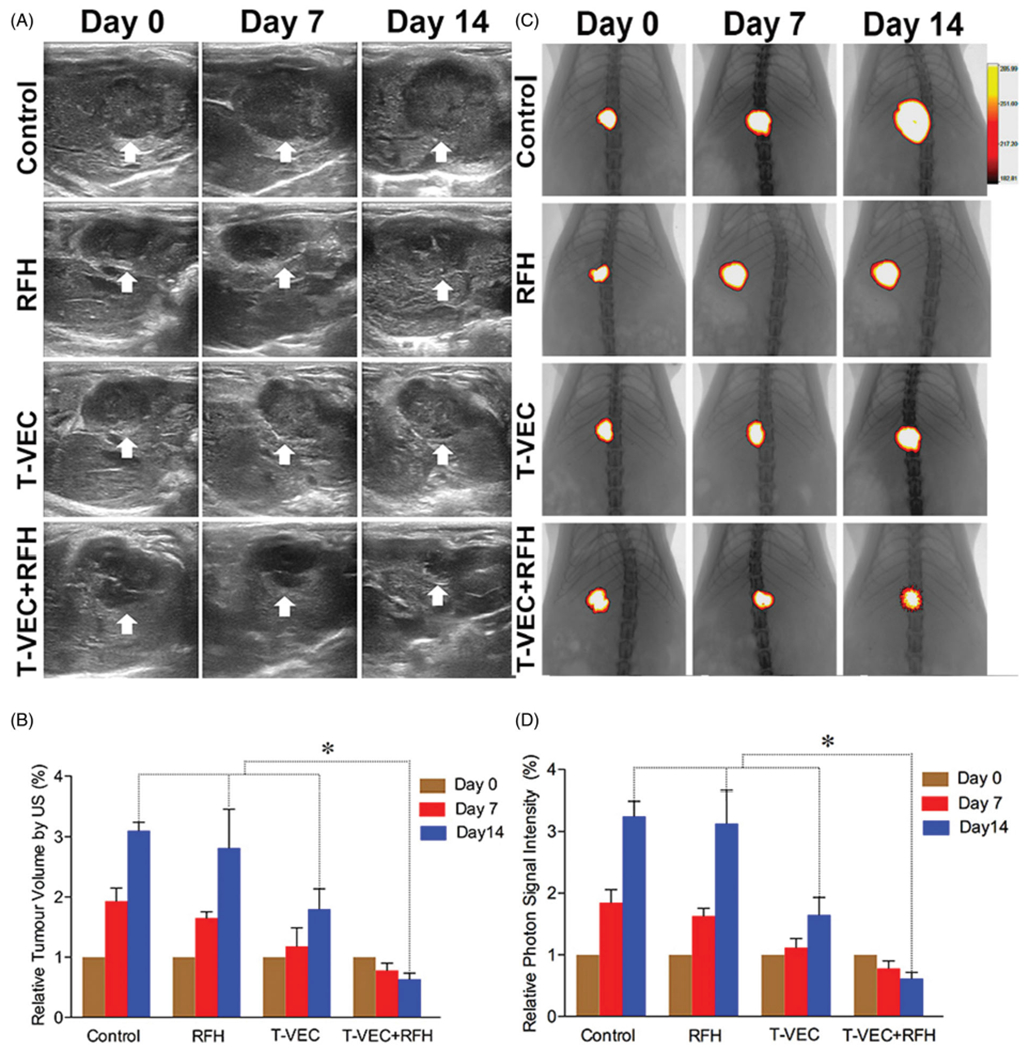
(A&B) Ultrasound imaging is used to follow up the tumor growth (arrows) at days 0, 7 and 14, showing a significant decrease in relative tumor volume with combination therapy compared with other three treatments (*n*=6/group, *p<.005). (C&D) Optical/X-ray imaging is used to follow up tumor responses to the treatments at days 0, 7 and 14, demonstrating a significant decrease in both relative fluorescent signal (golden-yellow color) and tumor size with combination therapy compared to other three treatments (*n*=6/group, *p<.05).

**Figure 5.**
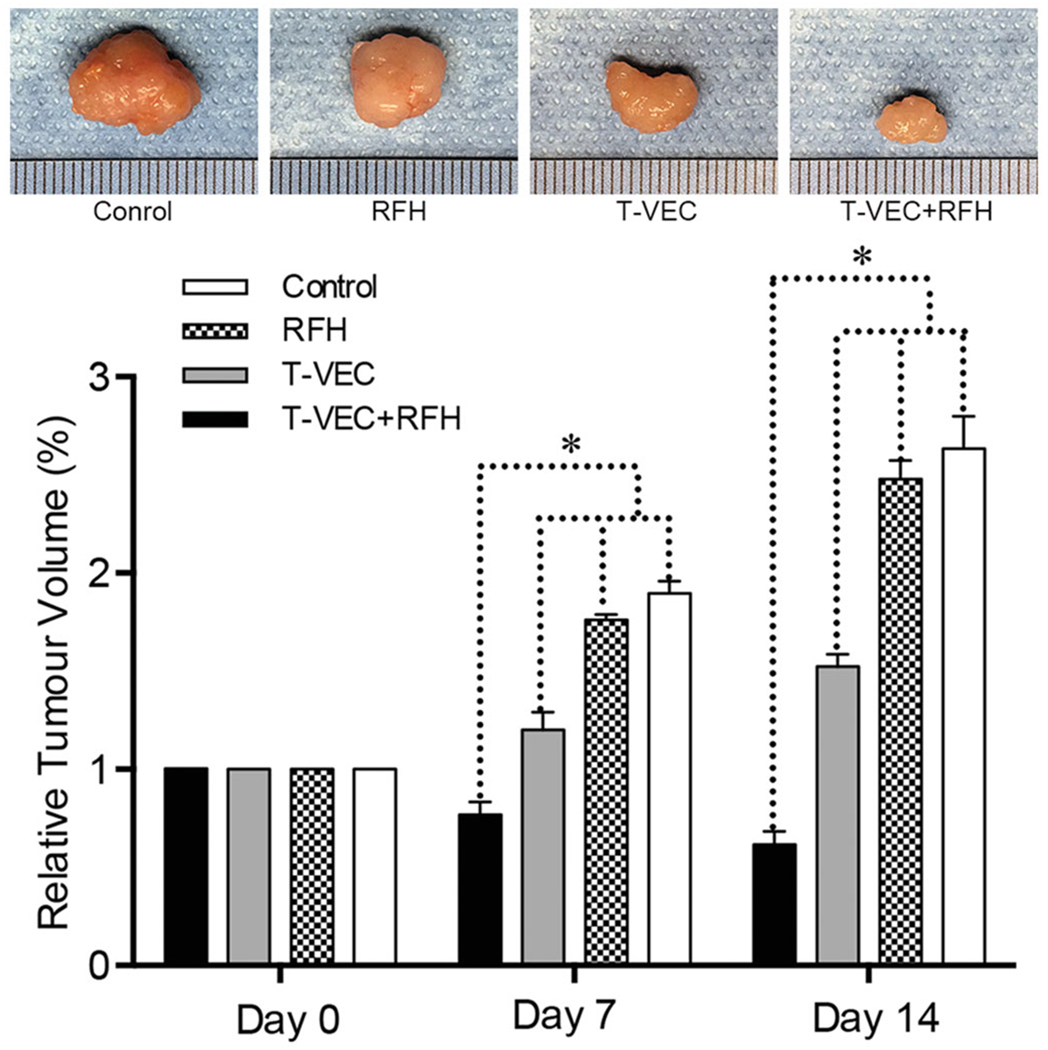
Representative tumors harvested from four different animal groups, demonstrating the smallest tumor size in the combination therapy group (T-VEC+RFH) compared to other three groups (*n*=6/group, *p<.05).

**Figure 6.**
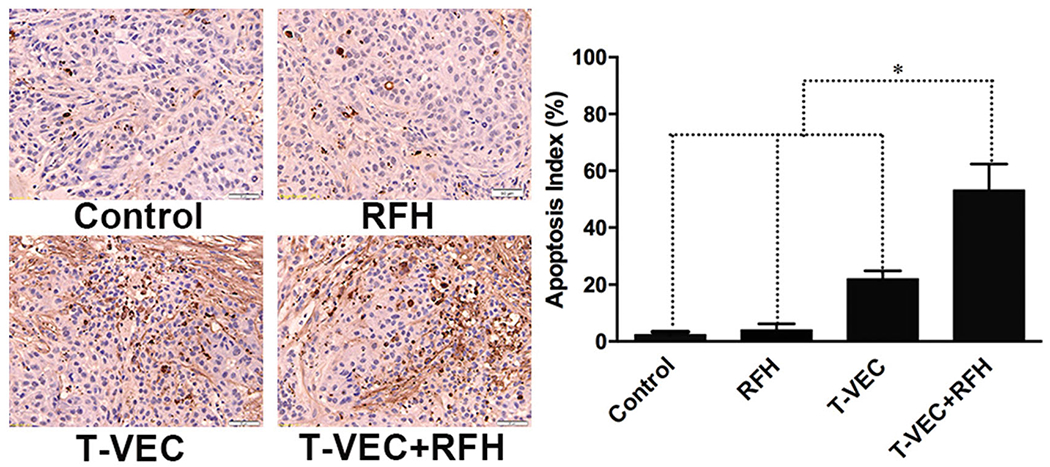
Apoptosis analysis using TUNEL staining further confirms more apoptotic cells (brown dots, 20×) in the combination therapy group than other three groups (*n*=6/group, *p<.001).

All animals survived after the experimental procedures without complications. Follow-up US images demonstrated the smallest relative tumor volume in the combination therapy group (0.63 ± 0.1, *n* = 6) compared with T-VEC-only group (1.8 ± 0.33, *n* = 6, *p* = .001), RF hyperthermia-only group (2.82 ± 0.64, *n* = 6, *p* = .002), and control group (3.1 ± 0.14, *n* = 6, *p* < .001) (Figure 4(A,B)). Optical imaging demonstrated significantly lower relative photon signal in the combination therapy group (0.62 × 10^7^ ± 0.1; *n* = 6) compared with that of T-VEC-only group (1.65 × 10^7^ ± 0.28; *n* = 6, *p* = .01), RFH-only group (3.13 × 10^7^ ± 0.53; *n* = 6, *p* < .001), and control group (3.25 × 10^7^ ± 0.24; *n* = 6, *p* < .001) (Figure 4(C,D)). Gross specimens were obtained at the end of the experiment, which revealed the smallest tumor size in the combination therapy group compared to other three groups (Figure 5). Histologic analysis of apoptosis by TUNEL staining further confirmed more apoptotic cells in the combination therapy group than the other three treatment groups (53.2 ± 0.9% vs 22.0 ± 2.9% vs 4.0 ± 2.2% vs 2.4 ± 1.0%, *n* = 6/group, *p*<.001) (Figure 6).

## Discussion

Although the ability of viruses to kill cancer cells has been recognized for nearly a century, clinical trials have just documented a therapeutic benefit in patients with cancer over the past decade [24–26]. Recent experiments have demonstrated the effectiveness of oncolytic viruses in the treatment of HCCs [27–30]. Wide clinical practice of radiofrequency ablation of small (<3cm) HCC’s has been shown to be highly efficacious, however, recurrence rates of larger HCC’s remains problematic. Given our previous groundwork in RFH combined with chemotherapy, we attempted to explore using RFH to enhance oncolytic virotherapy in treatment of HCC. The mechanism of hyperthermia-enhanced virotherapy may be attributed to heating-induced tissue fracture, enhanced T-VEC replication, accelerated tumor cell apoptosis, and consequently cytolysis of tumor cells [30–32].

We investigated delivering RFH immediately after intratumoral infusion of oncolytic virus into HCCs through a unique multi-functional perfusion-thermal RF electrode. Our study shows decreased survival of HCC cells in both *in vitro* experiments and in vivo experiments when combination therapy of oncolytic virotherapy and RFH is performed. Our study also demonstrates the utility of US and optical imaging in assessing response, which correlated well with pathologic analysis.

Our study has limitations. We applied a hyperthermia temperature at 42 °C and one dose of virus only. Further studies are necessary to investigate the synergistic effect of combining local oncolytic virotherapy at different doses with RFH at a higher temperatures similar to ablative temperatures used in clinical practice. Meanwhile, this project focused on establishing the “Proof-of-principle” that RFH can enhance destruction of hepatic tumor. Our next step is to validate the new technique on larger animal models such as rabbit, to specifically investigate the possibility of using RFH to specifically enhance the “cleaning” of cells/tissues at the tumor margin, and thereby to inhibit the recurrences and persistence after thermal ablation of larger tumors. Folllow-up was also limited to two weeks after the treatments. Longer durations would have resulted in orthotopic tumor masses in the control group greater than ten percent of the body weight, which is not approved by our institutional committees. In order to improve successful rate of orthotopic HCC models, we used nude rats with orthotopic HCCs. However, the nude rat model didn’t allow us to investigate the systemic immune response to T-VEC.

In conclusion, we have validated the feasibility of using image-guided RFH to enhance intratumoral high dose oncolytic virotherapy of HCC in nude rats, which is successfully monitored by US and optical imaging techniques. This combination technique offers great potential for improving local tumor control of larger HCCs and reducing systemic toxicity of virotherapy through intratumoral delivery of high-dose oncolytic viruses.

## References

[1] Ferlay J, Shin HR, Bray F, Estimates of worldwide burden of cancer in 2008: GLOBOCAN 2008. Int J Cancer. 2010;127:2893–2917. 21351269 10.1002/ijc.25516

[2] Bruix J, Sherman M, American Association for the Study of Liver Diseases. Management of hepatocellular carcinoma: an update. Hepatology. 2011;53:1020–1022. 21374666 10.1002/hep.24199 PMC3084991

[3] Borie F, Bouvier AM, Herrero A, Treatment and prognosis of hepatocellular carcinoma: a population based study in France. J Surg Oncol. 2008;98:505–509. 18932235 10.1002/jso.21159

[4] Llovet JM, Burroughs A, Bruix J. Hepatocellular carcinoma. Lancet. 2003;362:1907–1917. 14667750 10.1016/S0140-6736(03)14964-1

[5] Bruix J, Sherman M, Practice Guidelines Committee, American Association for the Study of Liver Diseases. Management of hepatocellular carcinoma. Hepatology. 2005;42:1208–1236. 16250051 10.1002/hep.20933

[6] Cho YK, Kim JK, Kim MY, Systematic review of randomized trials for hepatocellular carcinoma treated with percutaneous ablation therapies. Hepatology. 2009;49:453–459. 19065676 10.1002/hep.22648

[7] Chen MS, Li JQ, Zheng Y, A prospective randomized trial comparing percutaneous local ablative therapy and partial hepatectomy for small hepatocellular carcinoma. Ann Surg. 2006;243:321–328. 16495695 10.1097/01.sla.0000201480.65519.b8 PMC1448947

[8] Lau WY, Lai EC. The current role of radiofrequency ablation in the management of hepatocellular carcinoma: a systematic review. Ann Surg. 2009;249:20–25. 19106671 10.1097/SLA.0b013e31818eec29

[9] Livraghi T, Meloni F, Di Stasi M, Sustained complete response and complications rates after radiofrequency ablation of very early hepatocellular carcinoma in cirrhosis: is resection still the treatment of choice? Hepatology. 2007;47:82–89. 10.1002/hep.21933 18008357

[10] N’Kontchou G, Mahamoudi A, Aout M, Radiofrequency ablation of hepatocellular carcinoma: long-term results and prognostic factors in 235 Western patients with cirrhosis. Hepatology. 2009;50:1475–1483. 19731239 10.1002/hep.23181

[11] Ueno M, Hayami S, Shigekawa Y, Prognostic impact of surgery and radiofrequency ablation on single nodular HCC 5 cm: Cohort study based on serum HCC markers. J Hepatol. 2015;63:1352–1359. 26212030 10.1016/j.jhep.2015.07.013

[12] Kei SK, Rhim H, Choi D, Local tumor progression after radiofrequency ablation of liver tumors: analysis of morphologic pattern and site of recurrence. Am J Roentgenol. 2008;190:1544–1551. 18492905 10.2214/AJR.07.2798

[13] Takaki H, Yamakado K, Uraki J, Radiofrequency ablation combined with chemoembolization for the treatment of hepatocellular carcinomas larger than 5 cm. J Vasc Interv Radiol. 2009;20:217–224. 19097810 10.1016/j.jvir.2008.10.019

[14] Kaufman HL, Kohlhapp FJ, Zloza A. Oncolytic viruses: a new class of immunotherapy drugs. Nat Rev Drug Discov. 2015;14:642–662. 26323545 10.1038/nrd4663 PMC7097180

[15] Chiocca EA, Rabkin SD. Oncolytic viruses and their application to cancer immunotherapy. Cancer Immunol Res. 2014;2:295–300. 24764576 10.1158/2326-6066.CIR-14-0015 PMC4303349

[16] Harrington KJ, Puzanov I, Hecht JR, Clinical development of talimogene laherparepvec (T-VEC): a modified herpes simplex virus type-1-derived oncolytic immunotherapy. Expert Rev Anticancer Ther. 2015;15:1389–1403. 26558498 10.1586/14737140.2015.1115725

[17] Heo J, Reid T, Ruo L, Randomized dose-finding clinical trial of oncolytic immunotherapeutic vaccinia JX-594 in liver cancer. Nat Med. 2013;19:329–336. 23396206 10.1038/nm.3089 PMC4268543

[18] Zhang F, Le T, Wu X, Intrabiliary RF heat-enhanced local chemotherapy of a cholangiocarcinoma cell line: monitoring with dual-modality imaging-preclinical study. Radiology. 2014;270:400–408. 24471389 10.1148/radiol.13130866 PMC4228750

[19] Yin X, Yu B, Tang Z, Bifidobacterium infantis-mediated HSV-TK/GCV suicide gene therapy induces both extrinsic and intrinsic apoptosis in a rat model of bladder cancer. Cancer Gene Ther. 2013;20:77–81. 23258087 10.1038/cgt.2012.86

[20] Wang J, Shi Y, Bai Z, Radiofrequency hyperthermia-enhanced herpes simplex virus-thymidine kinase/ganciclovir direct intratumoral gene therapy of hepatocellular carcinoma. Int J Hyperthermia. 2016;1–8. 27569361 10.1080/02656736.2016.1229045 PMC5572512

[21] Du X, Qiu B, Zhan X, Radiofrequency-enhanced vascular gene transduction and expression for intravascular MR imaging-guided therapy: feasibility study in pigs. Radiology. 2005;236:939–944. 16040894 10.1148/radiol.2363041021

[22] O’Toole SA, Sheppard BL, McGuinness EP, The MTS assay as an indicator of chemosensitivity/resistance in malignant gynaecological tumours. Cancer Detect Prev. 2003;27:47–54. 12600417 10.1016/s0361-090x(02)00171-x

[23] Barltrop JA, Owen TC, Cory AH, 5-(3-carboxymethoxyphenyl)-2-(4, 5-dimethylthiazolyl)-3-(4-sulfophenyl) tetrazolium, inner salt (MTS) and related analogs of 3-(4, 5-dimethylthiazolyl)-2, 5-diphenyltetrazolium bromide (MTT) reducing to purple water-soluble formazans as cell-viability indicators. Bioorganic Med Chem Lett. 1991;1:611–614.

[24] Moore AE. The destructive effect of the virus of Russian Far East encephalitis on the transplantable mouse sarcoma 180. Cancer. 1949;2:525–534. 18131412 10.1002/1097-0142(194905)2:3<525::aid-cncr2820020317>3.0.co;2-o

[25] Moore AE. Effect of inoculation of the viruses of influenza A and herpes simplex on the growth of transplantable tumors in mice. Cancer. 1949;2:516–524. 18131411 10.1002/1097-0142(194905)2:3<516::aid-cncr2820020316>3.0.co;2-p

[26] Andtbacka RH, Kaufman HL, Collichio F, Talimogene laherparepvec improves durable response rate in patients with advanced melanoma. J Clin Oncol. 2015;33:2780–2788. 26014293 10.1200/JCO.2014.58.3377

[27] Song T-J, Eisenberg DP, Adusumilli PS, Oncolytic herpes viral therapy is effective in the treatment of hepatocellular carcinoma cell lines. J Gastrointest Surg. 2006;10:532–542. 16627219 10.1016/j.gassur.2005.08.036 PMC1444941

[28] Hernandez-Gea V, Alsinet C, Llovet JM. Oncolytic immunotherapeutic virus in HCC: Can it compete with molecular therapies? J Hepatol. 2013;59:882–884. 23673136 10.1016/j.jhep.2013.05.006

[29] Wang J, Xu L, Zeng W, Treatment of human hepatocellular carcinoma by the oncolytic herpes simplex virus G47delta. Cancer Cell Int. 2014;14:83. 25360068 10.1186/s12935-014-0083-y PMC4213511

[30] Eisenberg DP, Carpenter SG, Adusumilli PS, Hyperthermia potentiates oncolytic herpes viral killing of pancreatic cancer through a heat shock protein pathway. Surgery. 2010;148:325–334. 20633729 10.1016/j.surg.2010.05.005

[31] Chang E, Chalikonda S, Friedl J, Targeting vaccinia to solid tumors with local hyperthermia. Hum Gene Ther. 2005;16:435–444. 15871675 10.1089/hum.2005.16.435

[32] Jung B-K, Lee YK, Hong J, Mild hyperthermia induced by gold nanorod-mediated plasmonic photothermal therapy enhances transduction and replication of oncolytic adenoviral gene delivery. ACS Nano. 2016;10:10533–10543. 27805805 10.1021/acsnano.6b06530

